# Exploring timely and safe discharge from ICU: a comparative study of machine learning predictions and clinical practices

**DOI:** 10.1186/s40635-025-00717-z

**Published:** 2025-01-24

**Authors:** Chao Ping Wu, Rachel Benish Shirley, Alex Milinovich, Kaiyin Liu, Eduardo Mireles-Cabodevila, Hassan Khouli, Abhijit Duggal, Anirban Bhattacharyya

**Affiliations:** 1 Cleveland Clinic, 9500 Euclid Ave, Cleveland, OH 44195 USA; 2 Mayo Clinic, 4500 San Pablo Road, Jacksonville, FL 32224 USA

**Keywords:** Machine learning, Critical care, ICU discharge practice, Decision support in ICU discharge

## Abstract

**Background:**

The discharge practices from the intensive care unit exhibit heterogeneity and the recognition of eligible patients for discharge is often delayed. Recognizing the importance of safe discharge, which aims to minimize readmission and mortality, we developed a dynamic machine-learning model. The model aims to accurately identify patients ready for discharge, offering a comparison of its effectiveness with physician decisions in terms of safety and discrepancies in discharge readiness assessment.

**Methods:**

This retrospective study uses data from patients in the medical ICU from 2015-to-2019 to develop ML models. The models were based on dynamic ICU-readily available features such as hourly vital signs, laboratory results, and interventions and were developed using various ML algorithms. The primary outcome was the hourly prediction of ICU discharge without readmission or death within 72 h post-discharge. These outcomes underwent subsequent validation within a distinct cohort from the year 2020. Additionally, the models’ performance was assessed in comparison to physician judgments, with any discrepancies between the two carefully analyzed.

**Result:**

In the 2015-to-2019 cohort, the study included 17,852 unique ICU admissions. The LightGBM model outperformed other algorithms, achieving a AUROC of 0.91 (95%CI 0.9–0.91) and performance was held in the 2020 validation cohort (*n* = 509) with an AUROC of 0.85 (95%CI 0.84–0.85). The calibration result showed Brier score of 0.254 (95%CI 0.253–0.255). The physician agreed with the models’ discharge-readiness prediction in 84.5% of patients. In patients discharged by physicians but not deemed ready by our model, the relative risk of 72-h post-ICU adverse outcomes was 2.32 (95% CI 1.1–4.9). Furthermore, the model predicted patients’ readiness for discharge between 5 (IQR: 2–13.5) and 9 (IQR: 3–17) hours earlier in our selected thresholds.

**Conclusion:**

The study underscores the potential of ML models in predicting patient discharge readiness, mirroring physician behavior closely while identifying eligible patients earlier. It also highlights ML models can serve as a promising screening tool to enhance ICU discharge, presenting a pathway toward more efficient and reliable critical care decision-making.

**Supplementary Information:**

The online version contains supplementary material available at 10.1186/s40635-025-00717-z.

## Background

Despite a significant increase in intensive care unit (ICU) beds in hospitals across the United States, most regions still struggle with low ICU bed availability per capita and leading to increased occupancy rates [1]. ICUs are known to be resource-intensive and are responsible for a major portion of the hospital budget [2]. The flow of patients into the ICU has been improved through the use of early warning scoring systems, rapid response teams, and transition of care models [3]. However, the outflow of patients from the ICU depends on clinician decisions and institutional preferences [4].

The variability of clinician decision-making and the heterogeneity of clinical practice have led to major bottlenecks in the transfer of patients out of the ICU [5]. These delays in ICU discharge have significant downstream effects, including limited ICU bed availability for new patients, increased costs, and an increased patient length of stay [6]. The delays are driven by several factors, including the difficulty of predicting a patient’s readmission risk after discharge, the lack of decision-supporting tools, and the absence of system-based practice guidelines to facilitate clinical decision-making around ICU discharges [7]. Operational factors, such as bed availability and staffing constraints also complicate the process [8]. Attempts to improve this process through staffing increases or tele-ICU services are costly and still do not address the risk of variability [9].

Scoring systems developed for ICU admission are sometimes used to predict disease trajectory or recovery, but these systems have not been validated for safe discharge [7]. The readmission rate of ICU patients within 48 h is 2% [10], and higher in academic centers [6]. This underscores the need for better clinician support tools [7]. Machine learning has the potential to improve discharge prediction. However, prediction models that attempt to predict discharge based on characteristics at the time of admission have encountered several challenges, such as the temporality of disease trajectories, patient heterogeneity, and treatment effects [11]. Although dynamic models that incorporate the effect of time show promise [11, 12], no studies have compared the performance of any severity of disease systems with physician performance at the time of discharge from the ICU.

Our study aimed to develop ML models based on dynamic real-world electronic health records (EHR) that can accurately predict discharge readiness while assessing the safety of discharge by analyzing the composite outcome of readmission and mortality after discharge. Additionally, we compare the effectiveness of these ML-driven decisions with those made by physician-led teams.

## Methods

### Source of data and participants

A retrospective cohort consisting of all adult patients admitted to the Medical ICUs at Cleveland Clinic from January 1, 2015, to March 31, 2019, as the primary dataset. A separate cohort of patients admitted between April 1st, 2020, and May 31st, 2020, referred as a secondary dataset. Patients who were either discharged to palliative care, hospice, or were transferred to other ICUs, such as surgical ICU, cardiothoracic ICUs, were excluded from the analysis. The study was approved by the Institution Review Board.

### Outcomes

The primary outcome of our study was ICU discharge without adverse events within 72 h post-discharge. Adverse events were defined as either readmission or death. Readmission was defined as multiple ICU admissions during the same hospitalization. Therefore, an adverse event-free discharge entailed transfer to a regular nursing floor, long-term care facility, or home, without subsequent readmission or death.

### Predictors and feature engineering

The selection of variables was made based on both clinical expertise and a review of prior literature [13, 14]. These variables (as shown in e-Table 1 in supplement), were extracted from the EHR and included patient demographic information, comorbidities, common laboratory values, vital signs, medication use, and urine output. In addition, data on various ICU interventions such as the administration of continuous renal replacement therapy, vasopressors, and oxygen devices were included. To guarantee the data’s immediacy and relevance, we captured these variables using the most recent data available, with updates occurring at least hourly and, in some instances, daily. This approach involves employing the last observation carried forward method, where the maximum period considered for data recency extends back to the patient’s admission, ensuring the inclusion of essential variables recorded continuously from admission onwards. Detailed methods for preprocessing, including imputation and management of missing values (see e-Fig. 1 in supplementary), are documented in the supplementary material.

### Model development

For developing predictive ML models, we utilized the primary dataset from the 2015-to-2019 cohort. Our approach involved generating hourly predictions of the primary outcome through multivariable logistic regression (LR) and incorporating three machine learning algorithms: Random Forest (RF), Light Gradient Boosting Machine (LightGBM), and Neural Networks (NN). To mitigate overfitting, we partitioned the dataset into an 80% training set and a 20% testing set, further employing tenfold cross-validation within the training subset to strengthen model validation (see e-Fig. 2 in supplementary).

Hyperparameter tuning via grid search was conducted for all models to optimize their performance (see e-Table 2 in supplementary) with area under the receiver operator characteristic curve (AUROC), the result of the models was assessed using Delong’s method with significance level of 0.05. Calibration of the final model selected is measured with Brier score and plot calibration curve.

To assess the model drift, we evaluated the performance of these models on the secondary dataset—the 2020 cohort (see e-Fig. 2 in supplementary).

### Model and physician comparison

At our facility, physicians reviewed patients’ conditions and decided on ICU discharge eligibility daily at 8:30 a.m. This process focused on discharge readiness for existent patients or any new patients admitted overnight who were eligible for discharge based on all relevant information up to that point in time. Once the decision was made, the hospital administrative team initiated the transferring process, which comprised the majority of our ICU discharges. If a patient was initially considered ineligible for ICU discharge at 8:30 a.m. but later met the discharge criteria, the discharge process typically commenced following clinical rounds.

To compare physician and model prediction, we selected the best model developed in primary dataset and compared its predictions to those made by the physicians in the secondary dataset. In instances where the model and the physicians made different predictions, we conducted manual chart reviews to identify potential contributing factors to the discrepancies. These findings allowed us to evaluate the effectiveness of the model in relation to human professionals and assess its potential utility in the clinical setting.

### Statistical analysis and feature importance

We used mean values and standard deviations to summarize the results for continuous variables. To understand and illustrate the importance of features at both the global/entire population, and local/individual patient levels, we employed the Shapley additive explanation (SHAP) approach. In evaluating the performance of the prediction models, we used several metrics such as the AUROC, sensitivity, specificity, positive predictive value, and negative predictive value. We carried out all analyses using Scikit-Learn v0.21.3 and Python v.3.6.6.

### Role of funding sources

The study design, data collection, analysis, interpretation, report writing, and publication submission were not influenced by the funding source.

## Results

### Patient characteristics

During the study period, 12,809 unique patients were admitted to the Medical ICUs, with 17,852 unique admissions recorded in the primary dataset. The secondary dataset included 449 unique patients with 509 admissions (Table 1).The average age of patients in the two cohorts was 60.6 years and 61.7 years, respectively. The frequency of invasive mechanical ventilation was similar in both cohorts, with 30.5% of patients in the primary dataset and 30.8% of patients in the secondary dataset. (p = 0.261) The frequency of patients requiring renal replacement therapy was lower in the primary dataset compared to the secondary dataset (7.8% vs 12.8%, p < 0.001), while the frequency of patients requiring vasopressors was higher in the primary dataset compared to the secondary dataset (17.2% vs 13.0%, p = 0.014). Within 72 h after discharge, the mortality rate was also higher in the primary dataset compared to the secondary dataset (2.6% vs. 1.3%), while the readmission rate was similar between the two cohorts (6.8% vs. 6.5%).

**Table 1:** Patient characteristics in primary database (2015–2019 cohort) and secondary database (2020 cohort)

	Primary dataset: 2015–2019 cohort (*n* = 17,852)	Secondary dataset: 2020 cohort (*n* = 509)
Age, mean (SD)	60.6 (16.4)	61.7 (16.2)
Race: *n* (%)		
Caucasian	10,738 (60.2)	277 (54.4)
Black	6053 (33.9)	205 (40.3)
Asians	131 (0.7)	6 (1.2)
Other	881 (4.9)	19 (3.7)
Sex: female, *n* (%)	8654 (48.5)	219 (43.0)
Comorbidities: *n* (%)		
Diabetes	7061 (45.7)	172 (44.6)
Chronic kidney Disease	6482 (42.0)	178 (46.1)
Liver cirrhosis	2182 (14.1)	81 (21.0)
Congestive heart failure	4682 (30.3)	61 (15.8)
COPD,	4261 (27.6)	31 (8.0)
Malignancy	217 (1.4)	
Admission lab value: mean (SD)		
Blood urea Nitrogen (mg/dL)	31.5 (24.6)	33.8 (27.5)
Chloride (mg/dL)	100.1 (6.7)	101.0 (6.6)
Hemoglobin (g/dL)	10.1 (2.4)	10.0 (2.6)
Potassium (mmol/L)	4.2 (0.6)	4.2 (0.6)
Sodium (mmol/L)	137.9 (5.5)	137.7 (5.8)
Platelet (k/μL)	202.2 (114.5)	199.4 (117.8)
Admission vitals:		
Mean arterial pressure (mmHg)	84.2 (15.6)	83.9 (14.6)
Pulse rate (beats/min)	87.4 (17.3)	87.7 (17.0)
SpO2 (%)	96.4 (3.7)	96.3 (3.3)
Respiratory rate (times/min)	22.2 (6.0)	21.8 (5.4)
Temperature (°C)	98.2 (1.0)	98.1 (1.0)
Input output (ml/h),	39.0 (220.9)	-20.9 (258.1)
Mechanical ventilation, *n* (%)	5439 (30.5)	157 (30.8)
Require renal replacement therapy, *n* (%)	1398 (7.8)	65 (12.8)
Require pressors, *n* (%)	3077 (17.2)	66 (13.0)
Adverse outcomes at 24/48/72 h:		
Readmission, *n* (%)	407/590/724 (2.4/3.6/4.4)	11/21/25, (2.4/4.5/5.4)
Death, *n* (%)	225/337/431 (1.3/2.1/2.6)	1/3/6, (0.2/0.6/1.3)
All adverse outcomes, *n* (%)	625/903/1106 (3.83/5.5/6.8)	12/24/30 (2.6/5.2/6.5)

### Model performance

**Fig. 1:**
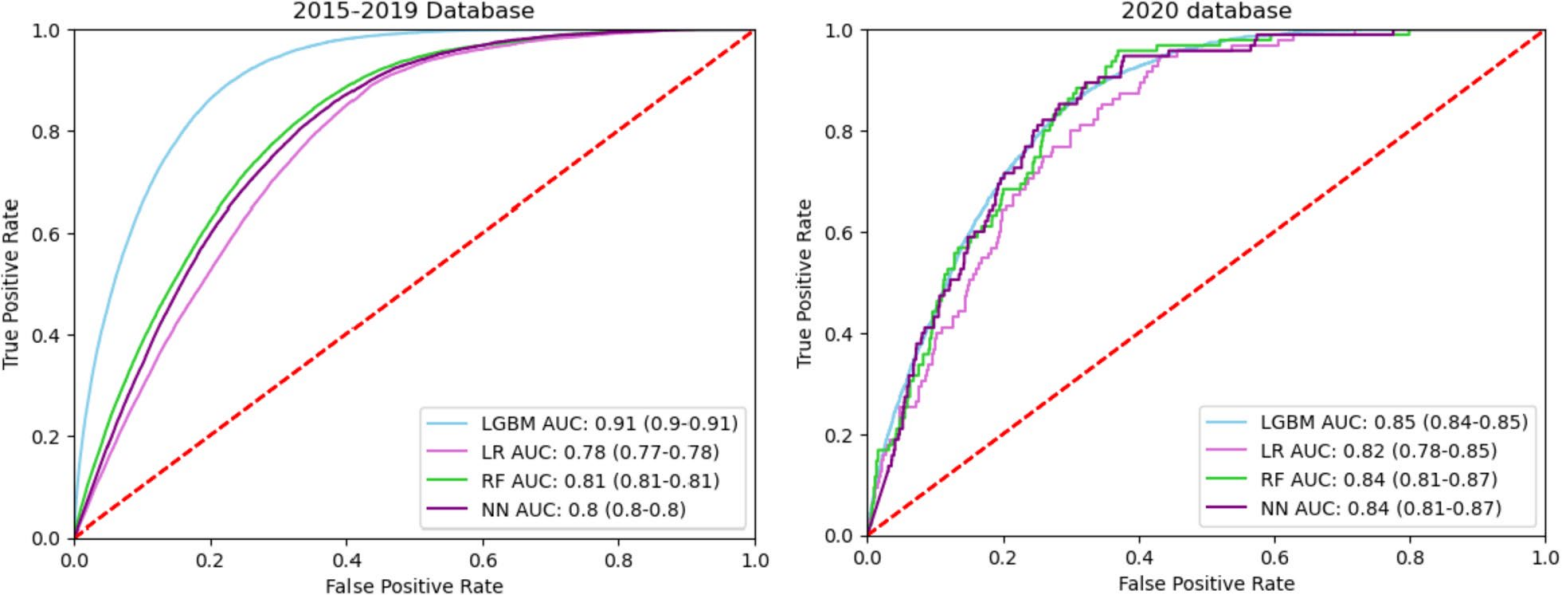
Performance of discharge prediction demonstrated with receiver operating characteristic curves of the four different algorithms, 2015–2019 database and secondary validation database in 2020. AUC = area under the curve; LGBM = light gradient-booster-machine; RF = random forest; NN = neural network

We evaluated the performance of four predictive models on the primary and secondary dataset. In the primary dataset (2015–2019 cohort), the results showed the AUROCs of LightGBM, RF, NN, and LR models were 0.91 (95% confidence interval [CI] 0.9–0.91), 0.81 (95% CI 0.81–0.81), 0.8 (95% CI 0.8–0.8), and 0.78 (95% CI 0.77–0.78), respectively. For the secondary dataset (2020 cohort), the LightGBM model demonstrated the highest AUROC at 0.85 (95% CI 0.84–0.85), which was significantly more robust compared to the AUROCs of the RF (0.84, 95% CI 0.81–0.87), NN (0.84, 95% CI 0.81–0.87), and LR (0.82, 95% CI 0.78–0.85) models (Fig. 1).

Consequently, the LightGBM model was selected for further analysis due to its performance and stability. In the calibration curve plot (see e-Fig. 3), our model exhibited a slope of 0.90 and an intercept of 0.005, indicating a slight underestimation of probabilities. The Brier score was 0.254 (95% CI 0.253–0.255), reflecting good calibration. At a probability threshold of 0.90, the LightGBM model demonstrated a specificity of 0.871 in predicting discharge, along with sensitivity, accuracy, positive predictive value, negative predictive value, and F1 score of 0.70, 0.86, 0.21, 0.98, and 0.32, respectively (e-Table 3).

**Fig. 2:**
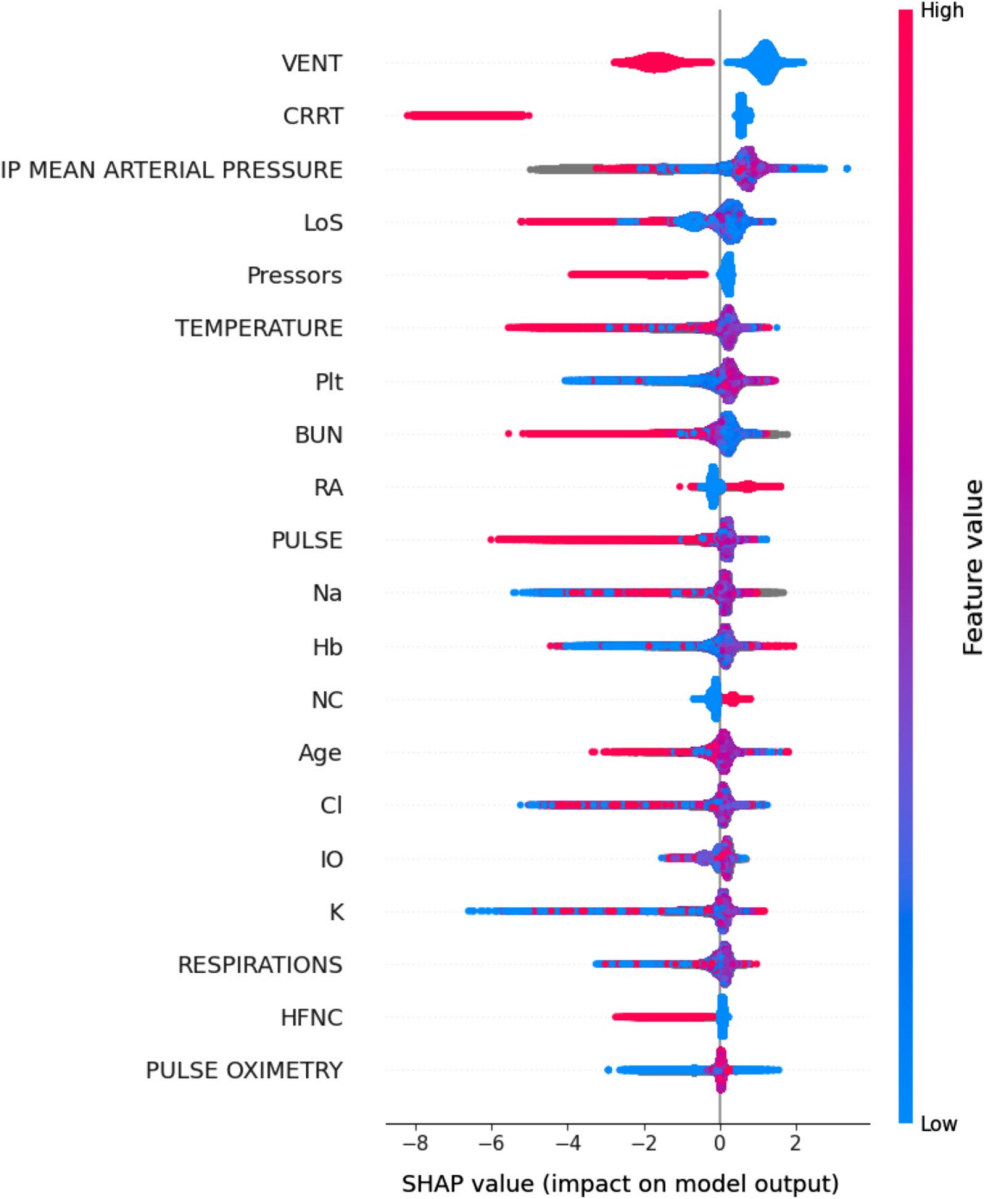
Top 10 most important features in the training dataset in the primary analysis. Vent: ventilator, CRRT: continuous renal replacement therapy, IP MEAN ARTERIAL PRESSURE: mean arterial pressure, RA: breathing under room air, LoS: length of stay in the ICU, NC: breathing with nasal cannula, Pressors: whether patient is on vasopressors, IO: input and outputs, BUN: blood urea nitrogen, PULSE: pulse rate, Hb: hemoglobin, TEMPERATURE: body temperature, Plt: Platelet, RESPIRATIONS: respiratory rate, HFNC: breathing with high-flow nasal cannula, PEEP device: breathing with positive end expiratory pressure devices, PULSE OXIMETRY: saturation percentage at pulse oximeter

The LightGBM model identified several important features that influenced the prediction of a patient’s eligibility for discharge from the ICU. As shown in Fig. 2, the top two features were the use of invasive mechanical ventilators and continuous renal replacement therapy. In addition, the dependence plot indicated that extreme vitals, such as blood pressure, pulse rate, and respiratory rate, were associated with a lower probability of discharge (e-Fig. 4). These findings are consistent with clinical practice and are further supported by the explainability of the model through the dependence plot and summary plot.

### Comparison of discharge prediction results between the LightGBM model and physician decisions

In the 2020 cohort’s secondary dataset consisting of 1986 patient-days, the concordance rate between the discharge decisions made by the model and physicians was 84.5% (Table 2). Despite this high concordance rate, there were some instances of discordance. Specifically, in 187 patient-days (9.4%), the model predicted a high probability of discharge, but the physician decided to keep the patient in the ICU, while in 120 patient-days (6%), the model predicted a low probability of discharge, but the physician decided to discharge the patient.

**Table 2:** In total 1986 patients, there was concordance in 1679 (84.5%) of patient-days between physician and model prediction for discharge

	Physician prediction	
Model prediction:	No discharge	Discharge
No discharge	1590 (80.1%)	120 (6%)
Discharge	187 (9.4%)	89 (4.5%)

There were 307 patient-days (15.5%) where the prediction from the physician and model were discordant

Patients who were discharged from the ICU by the physician-led team, in discordance with the model’s prediction of not being discharge-ready, had a higher risk of adverse outcomes. Specifically, these patients had a relative risk of 2.01 (95% CI 0.88–4.58) for adverse outcomes within 48 h and 2.23 (95% CI 1.1–4.9) within 72 h post-discharge, compared to patients whom the model predicted were ready for discharge (see e-Table 4 in supplementary). Additionally, the machine learning model demonstrated its efficacy in identifying patients as discharge-ready on average 5 h earlier (IQR: 2–13.5) with a discharge probability threshold of 0.9, and 9 h earlier (IQR: 3.0–17.0) with a threshold of 0.85, compared to physician assessments (e-Fig. 5).

A manual chart review was conducted on a sample of 307 patient-days in the patients who were not discharged from the ICU but predicted as discharge-ready by our ML model. Out of these patients, 193 (63%) patient-days had medically stable patients suitable for discharge from the ICU, and most of these patients were ultimately discharged on the day of prediction. In addition, 61 other patient-days (20%) had patients who required extended stays in the ICU for procedures such as endoscopy, otorhinolaryngology fiberscope examination, or goals of care discussions. These patients, who had stable vital signs and laboratory values, were predicted as discharge-ready by the ML model (Table 3).

**Table 3:** Chart review results in 307 patient-days with prediction discrepancies between physicians and models

	Total	
1. Clinical necessity to stay in the ICU	61	20%
Pending procedure	21	
Pending consult	5	
Pending GOC	5	
Non-pressor medication (e.g., Nicardipine ggt, Nitro ggt)	7	
Clinical changes (e.g., new fever, Afib, AMS, delirium)	17	
Other	6	
2. Data collection error	25	8%
High oxygen requirement at the time of prediction (HFNC, BiPAP)	10	
On CRRT at the time of prediction	9	
On pressors at the time of prediction	8	
3. Physician comfort zone*	28	9%
Monitor vitals (e.g., BP)	15	
Off pressors < 24 h	2	
Extubate < 24 h	7	
Abnormal lab (e.g., Na < 120)	4	
4. Medically ready	193	63%
Medically ready, pending bed availability	25	
Discharge later that day	178	
	307	100%

### Interpretable interface

**Fig. 3:**
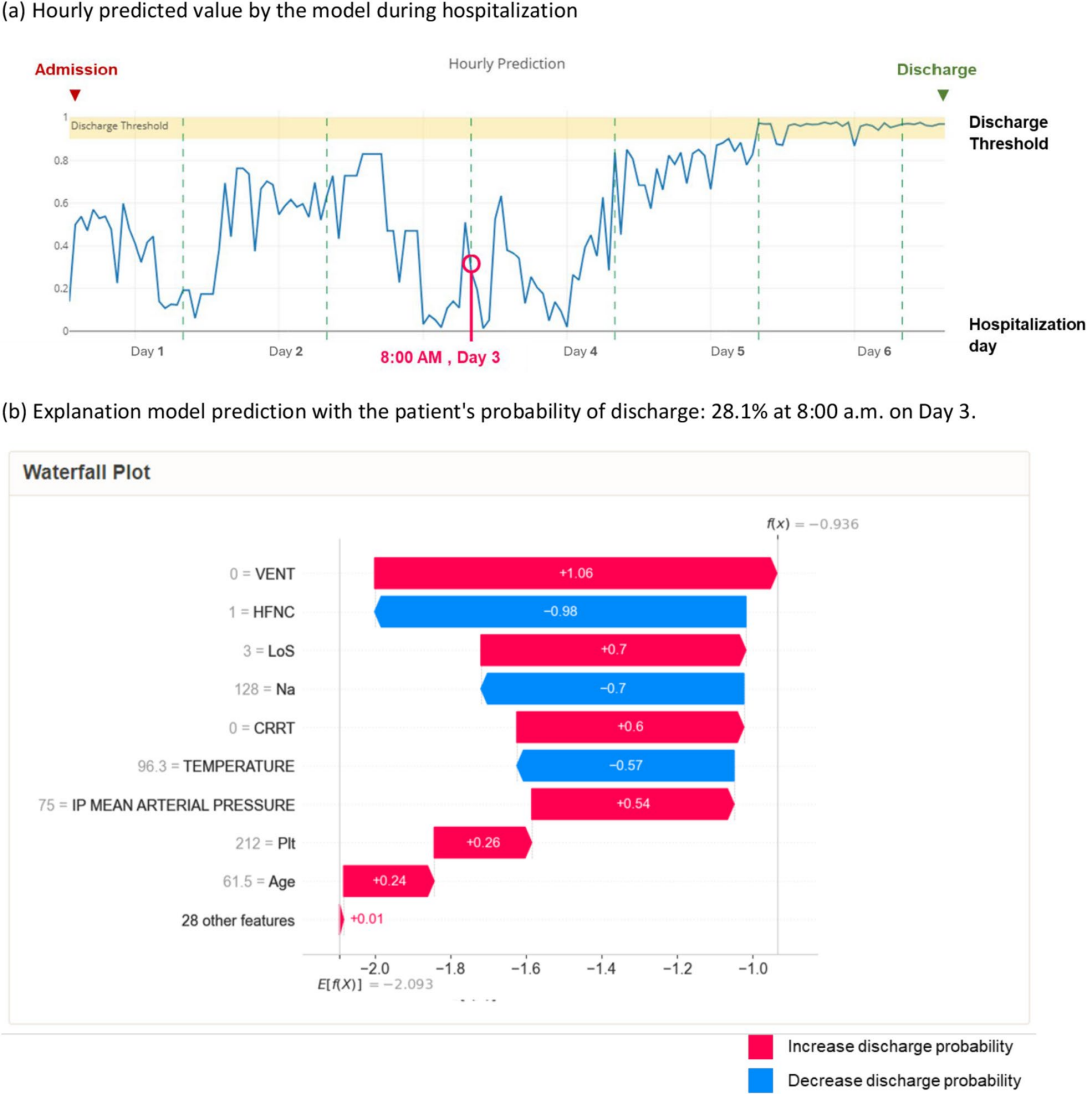
Case study of individual prediction. **a** A 61.5-year-old female with a history of COPD, cirrhosis, and diabetes presented to the ICU with acute hypoxic respiratory failure secondary to rhinovirus infection. Figure illustrates the hourly predictive probability of discharge for this patient. At 8am of third day of admission, the LightGBM model estimated a 28.1% probability of discharge (represented by the pink circle), which was below the discharge threshold. Subsequently, the patient’s discharge probability increased at 8am on day 5 and remained stable thereafter. The patient was ultimately discharged from the ICU to the regular nursing floor at 3 pm on day 6, with a predicted discharge probability of 96.9% according to the model. **b** The waterfall plot, visualizes prediction interpretation by ranking features according to their significance at 8:00 am on day 3 of admission. It highlights that the absence of invasive mechanical ventilation is a strong predictor of discharge, an effect that is mitigated by the utilization of high-flow nasal cannula

To better understand the model’s predictions and apply them to individual patients, we developed an interface to facilitate interpretation. In the case study, we applied the model to evaluate the hospitalization course of a patient with acute hypoxic respiratory failure who required high-flow nasal cannula (HFNC) when admitted. As the patient’s condition improved and was transitioned to nasal cannula, the ML model’s probability of discharge increased (shown in e-Fig. 6). At each timestamp, the interface depicted various factors that influenced the prediction in the waterfall plot. The prediction is the result of various factors, represented by pink and blue arrows, listed in order of their effect on the probability of discharge from ICU. Factors indicated by pink arrows increase this probability, while those indicated by blue arrows decrease it. In the case study, the top three factors influencing the prediction were absence of mechanical ventilation (VENT = 0), use of HFNC, and length of stay (LoS = 3) as shown in Fig. 3.

## Discussion

This research plays a crucial role in addressing a pressing need to streamline the ICU discharge process and improve patient outcomes via algorithmic approaches. Using 26 commonly recorded ICU features, our LightGBM model accurately identified the discharge-readiness of patients from the ICU while maintaining a similar adverse event rate as physician-led discharges. Depending on the threshold selected, we observed that our model was able to flag these patients at least 5–9 h before the physician-led teams identified these patients. We explored with a variety of thresholds and observed a continuum of improvement in the model’s prediction time. Our chosen thresholds of 0.85 and 0.9 had performances that mimicked the physician-led team’s performance while providing meaningful lead times for throughput planning. For patients that were discharged by the physicians but predicted non-discharge-ready by our model, the incidence of adverse events within 72 h of discharge was 7 times higher. In general, physicians and our model agreed over 80% of the time. In less than 10% of all cases, the model predicted discharge-ready at 8:00 am while physicians held back the discharge. Among these patients, two thirds were eventually discharged later the same day, while majority of the remaining patients stayed in the ICU due to expected procedures or consultations and not because of a clinically unstable state. When interpreting the important features that affected the model’s decision and thresholds used by our model, it was consistent with what is considered a valid reason for staying in the ICU. Use of mechanical ventilation, continuous renal replacement therapy, and features measuring hemodynamic instability were the most important features that influenced our model’s decisions.

Despite the universal occurrence of ICU discharge, our literature review found fewer than 40 reported studies on discharge prediction [11, 13–18] (see supplementary for literature search method). Most of these models are retrospective in nature, often derived from features and values from day of admission thus unable to capture the temporality in a patient’s ICU course. Additionally, several of these studies were conducted on publicly available databases that made interpretation to the local context and model generalizability difficult. To address these challenges, we trained and tested the algorithm using real-world patient EHR data from our ICUs and tailored the algorithm to suit our institution’s needs and adapt to the local context. We designed our model to function dynamically, thus capturing the status of the patient and predicting their discharge probability in a continuous fashion. To ensure the accuracy of our algorithm, we rigorously checked for overfitting through analytical discipline. Furthermore, we validated our algorithm in the same ICU at a different time-period to assess longitudinal concept drift and address any concerns. We found that our model maintained comparable performance over time. Another limitation in many of the existing models is the lack of explainability. In our study, we have put significant effort into understanding how the variables add to the predictive value and also why a model would recommend in a certain way. We employed various methods to achieve this, including SHAP values for feature ranking, verifying that cutoff thresholds of these features were in line with what is clinically accepted and to simulate the impact of changing these clinical variables’ effect on each patient’s chances of successful discharge (e-Fig. 6) [19].

What sets our study apart from others is our exploration into the consequences of our model’s decisions compared to those made by physicians. Given the absence of concrete discharge criteria, ICU discharge remains a complex and subjective procedure, primarily dependent on the physician’s discretion [7]. This ambiguity results in diverse outcomes and an arduous process that resists algorithmic simplification [20]. The Society of Critical Care Medicine’s special article acknowledging these challenges while formulating ICU discharge guidelines has previously advocated for an algorithmic approach to identify suitable patients more objectively [7]. Our rigorous methodology enabled our model to successfully navigate these obstacles and match human performance levels. We confirmed this by directly comparing our model’s decisions with those of physicians, a comparison few studies have undertaken.

Our study does have some limitations. One of the main limitations is that it was retrospective and took place in only medical ICUs. This could make it difficult to apply our findings more broadly. But even with this limitation, our study showed that machine learning models can adjust to different behaviors of clinicians. To make our model more applicable to different settings, it may be necessary to retrain or refit it. Future research should consider applying our model across a diverse range of ICUs to increase its generalizability and robustness. Furthermore, exploring other potential applications of the model within critical care could be valuable. Our study strategically concentrated on the use of dynamic snapshot data to discern discharge eligibility at particular moments, as opposed to predicting forward. Nonetheless, we developed a tool that allows for the manipulation of feature values to observe their impact on discharge readiness, aiding in pinpointing factors that may impede discharge and offering insights for streamlining the discharge process. Additionally, we acknowledge the limitation related to the evaluation of our model’s prediction stability [21, 22], absence of causal inferential prediction, emphasizing the need for ongoing research to ensure model reliability and to advance causal inference research for achieving “actionable AI” [23].

Our study underscores the potential that algorithm-based risk stratification holds for early patient identification and enhanced discharge practice. By dissecting our model’s decision-making process and contrasting it with physicians’ judgments, we strengthen the credibility of our algorithm, therefore suggesting a promising pathway toward integrating more advanced and reliable decision-support systems into critical care. From a clinical standpoint, incorporating this model could potentially streamline physicians’ workflow, allowing more efficient allocation of critical care resources. By facilitating timely and accurate discharge, patient throughput could be improved without compromising patient safety, significantly impacting the quality of ICU care.

## Conclusion

Our study has demonstrated the remarkable potential of a machine learning model, trained on real-world electronic health records, as a highly effective screening tool for expediting the discharge eligibility assessment process among a diverse range of ICU patients. By providing throughput teams with a decision support tool that outperforms traditional methods, our model not only enhances efficiency, but also optimizes resource allocation in critical care settings. Its flexibility and adaptability, combined with the integration of clinical insights from physicians, make it a versatile approach that can be seamlessly applied across various clinical and hospital settings. This successful integration of machine learning into ICU operations highlights its transformative role in elevating healthcare outcomes and underscores its value as an indispensable tool in healthcare improvement efforts.

## Supplementary Information

Additional file 1.

## Data Availability

The datasets generated and/or analyzed during the current study are not publicly available due to Institutional Review Board (IRB) regulations and patient data confidentiality, but are available from the corresponding author on reasonable request.

## Abbreviations

AUROC: Area under the receiver operator characteristic curve
CI: Confidence interval
EHR: Electronic health record
ICU: Intensive Care Unit
IQR: Interquartile range
LR: Logistic regression
ML: Machine learning
NN: Neural networks
RF: Random Forest
SHAP: Shapley additive explanation
