# Supplementary material for: Exploring timely and safe discharge from ICU: a comparative study of machine learning predictions and clinical practices
Source: Intensive Care Med Exp. 2025 Jan 24;13:10. doi: 10.1186/s40635-025-00717-z (PMC11759737; doi:10.1186/s40635-025-00717-z)
Supplement: Supplementary file 1 — Additional file 1. [file 40635_2025_717_MOESM1_ESM.docx]

Supplemental content

**Ensuring Timely and Safe Discharge from ICU: A Comparative Study of Machine Learning Predictions and Clinical Practices**

Chao Ping Wu, MD^1^; Rachel Benish Shirley PhD^1^; Alex Milinovich BA^1^; Kaiyin Liu, MD^1^, Eduardo Mireles-Cabodevila, MD^1^; Hassan Khouli, MD^1^; Abhijit Duggal, MD^1^; Anirban Bhattacharyya, MD^2^.

*Affiliations*

1. Cleveland Clinic 9500 Euclid Ave, Cleveland, OH 44195, USA
2. Mayo Clinic 4500 San Pablo Road Jacksonville, FL 32224, USA

Contents

[Feature Engineering 2](#_Toc160605596)

[Missing Value and Imputation methods 3](#_Toc160605597)

[Model training 5](#_Toc160605598)

[Hyperparameter Tuning 6](#_Toc160605599)

[Model Performance 8](#_Toc160605600)

[Discharge prediction 8](#_Toc160605601)

[Adverse event prediction 8](#_Toc160605602)

[Model Calibration 9](#_Toc160605603)

[Explainability analysis 10](#_Toc160605604)

[User interface 11](#_Toc160605605)

[Literature Search 14](#_Toc160605606)

# Feature Engineering

**e-Table 1.** Input variables for machine learning models to predict discharge.

| Variables |  | Data type |
| --- | --- | --- |
| Age |  | Numeric |
| Race | Caucasian, Black, Asians, Other | Categorical |
| Gender | Male, Female | Categorical |
| Comorbidities | DM, CKD, Cirrhosis, COPD, CHF, Malignancy | Binary |
| Lab variables | Blood urea nitrogen, Chloride, Hemoglobin, Potassium, Sodium, Platelet | Numeric |
| Clinical variables | Pulse rate, Respiratory rate, Temperature, Blood pressure, Pulse oximeter, Urine Inputs and outputs | Numeric |
| Oxygen device | Room air, Nasal Canula, Non-Rebreather, Venti Mask, BiPAP, High flow nasal canula, Invasive mechanical ventilator, tracheostomy | Binary |
| ICU specific treatments | Continuous renal replacement therapy, Vasopressors | Binary |
| Length of Stay |  | Numeric |

## Missing Value and Imputation methods

To address missing values, given the nature of our data, which includes variables updated on an hourly or daily basis, such as vital signs, laboratory values, medication administration, and ICU interventions, we adopted the last observation carried forward (LOCF) method for imputation of missing values.

**Rationale**

We adapted this method based on its suitability for time-series and longitudinal data frequently encountered in healthcare settings, particularly in ICU where patient conditions are monitored closely, and data is recorded at regular intervals. This method assumes that the most recent observation remains a valid representation of the patient's status until a new observation is made. This assumption aligns with clinical practice in ICUs, where rapid changes in patient condition are closely monitored, and the latest data points are often the most relevant for clinical decision-making.

**Implementation of LOCF for Missing Data Imputation**

**Identification of Missing Data**: Our first step was to systematically identify missing values across the dataset for variables subject to LOCF imputation. This included patient demographic information, comorbidities, common laboratory values, vital signs, medication use, urine output, and data on ICU interventions.

**Application of LOCF**: For each variable with missing data, we traced back to the last available non-missing value for that specific patient and carried this value forward to fill the missing data point. This procedure was applied consistently across the dataset, ensuring that each missing value was replaced with the most recent available observation for that variable.


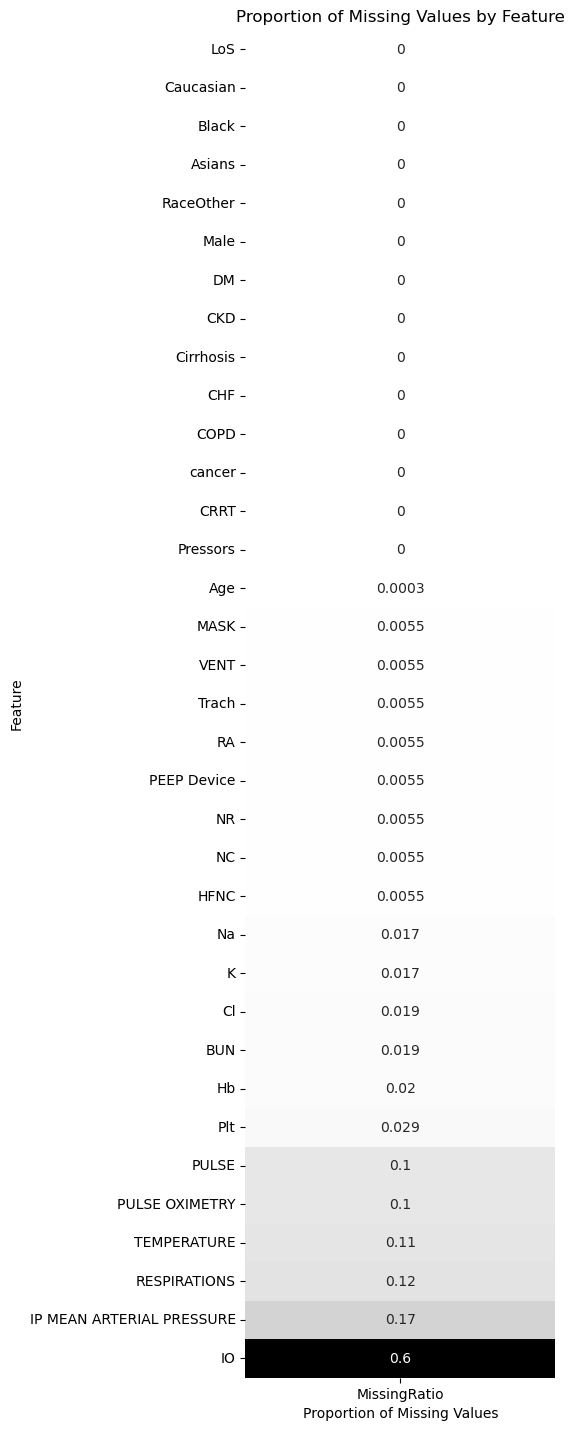


**e-Figure 1**: Missing data ratio after LOCF imputation method

**Data Integrity Checks**: Following imputation, we conducted checks to ensure data integrity and consistency. This included verifying that no future observations were inadvertently used and that the temporal order of data points was preserved.

# Model training

We used the primary dataset, the 2015-to-2019 cohort, to develop predictive ML models. To prevent overfitting, the dataset was randomly divided and stratified by unique admissions into training data (80%) and testing data (20%) using 10-fold cross-validation. We constructed predictive models using multivariable logistic regression (LR) and three ML algorithms: random forest (RF), Light Gradient Boosting Machine (LightGBM), and neural networks (NN). Hyperparameter tuning via grid search was conducted for all models to optimize their performance using primary dataset. To assess the model drift and generalizability, we evaluated the performance of these models on the secondary dataset – the 2020 cohort.

**e-Figure 2**: Overview of model building process


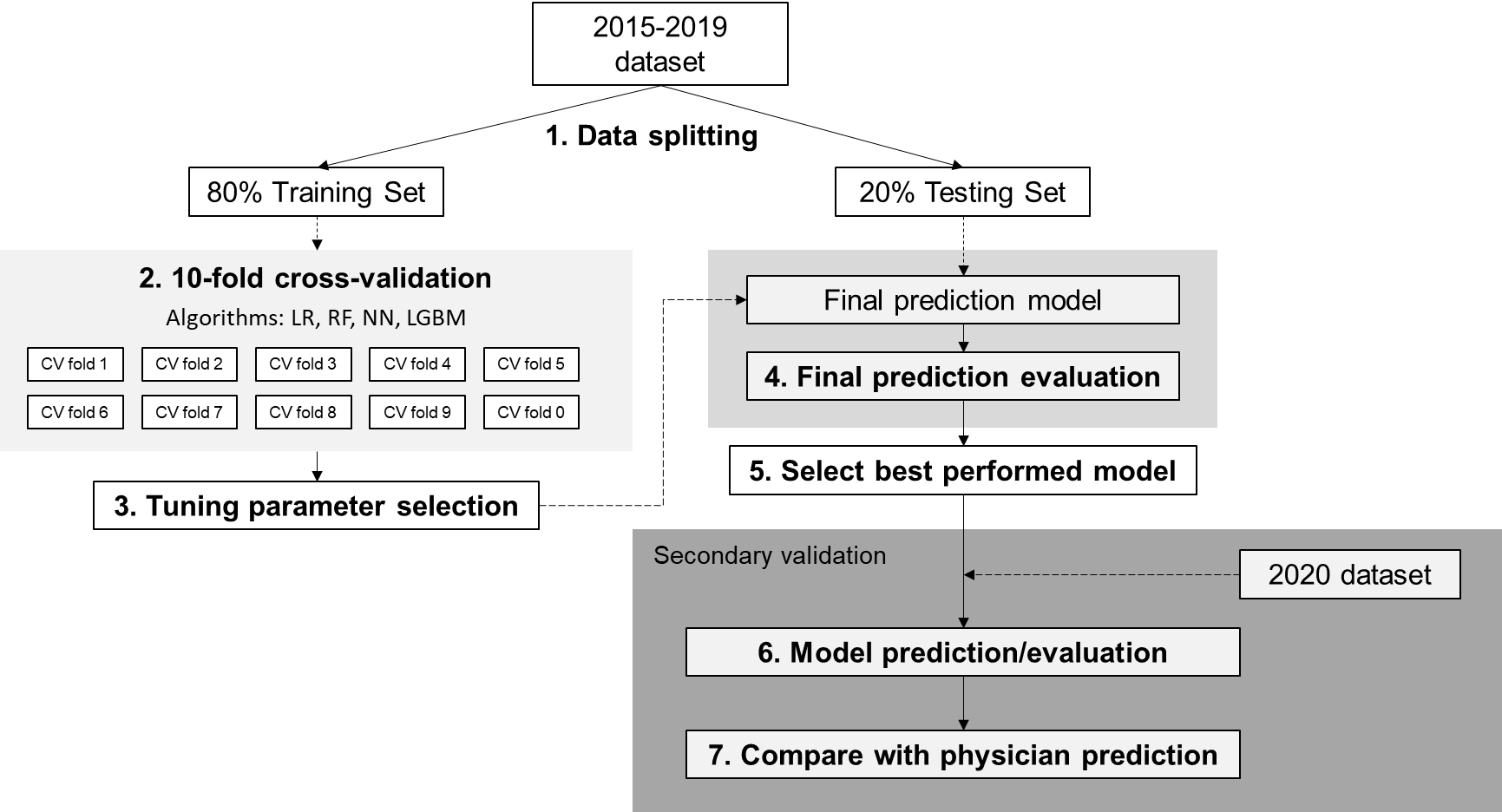


# Hyperparameter Tuning

**e-Table 2(a)**: Parameter Tuning Variants for Grid search across Machine Learning Algorithms

| **Algorithm** | **Parameter** | **Values used** |
| --- | --- | --- |
| **LR** | C | 0.01, 0.1, 1, 10 |
|  | penalty | 'l1', 'l2' |
| **RF** | n_estimators | 100, 200, 500 |
|  | max_depth | 10, 20, 30 |
|  | min_samples_leaf | 2, 4 |
|  | max_features | 'auto', 'sqrt' |
| **LightGBM** | learning_rate | 0.001, 0.01, 0.1, 0.2 |
|  | n_estimators | 100, 200, 500 |
|  | max_depth | -1, 10, 20 |
|  | num_leaves | 31, 50, 100 |
| **NN** | learning_rate | 0.001, 0.01 |
|  | epochs | 50, 100, 200 |
|  | batch_size | 32, 64, 128 |
|  | layers | [hidden layers configuration varies] |
|  | activation | 'relu', 'sigmoid' |

**e-table 2(b):** Optimized Parameters for Best Model Performance Across Algorithms

| **Algorithm** | **Parameter** | **Best Performed Model's Parameter** |
| --- | --- | --- |
| **LR** | C | 0.1 |
|  | penalty | 'l1' |
| **RF** | n_estimators | 500 |
|  | max_depth | 30 |
|  | min_samples_leaf | 2 |
|  | max_features | 'auto' |
| **LightGBM** | learning_rate | 0.01 |
|  | n_estimators | 200 |
|  | max_depth | 20 |
|  | num_leaves | 31 |
| **NN** | learning_rate | 0.01 |
|  | epochs | 100 |
|  | batch_size | 64 |
|  | layers | [hidden layers configuration varies] |
|  | activation | 'relu' |

# Model Performance

## Discharge prediction.

**e-Table 3: Threshold sensitivity analysis:** Model performance of LightGBM model in the primary dataset (2015-2019 cohort) over a range of probability threshold (AUC = 0.9)

| **Threshold** | **Sensitivity** | **Specificity** | **Positive Predictive value** | **Negative Predictive value** | **Accuracy** | **F1 score** |
| --- | --- | --- | --- | --- | --- | --- |
| 0.60 | 0.979 | 0.673 | 0.125 | 0.998 | 0.686 | 0.221 |
| 0.70 | 0.960 | 0.715 | 0.139 | 0.997 | 0.726 | 0.242 |
| 0.75 | 0.942 | 0.740 | 0.147 | 0.996 | 0.749 | 0.255 |
| 0.80 | 0.909 | 0.772 | 0.160 | 0.994 | 0.778 | 0.272 |
| 0.85 | 0.844 | 0.813 | 0.177 | 0.991 | 0.815 | 0.293 |
| 0.90 | 0.701 | 0.871 | 0.207 | 0.984 | 0.864 | 0.319 |
| 0.95 | 0.333 | 0.958 | 0.273 | 0.968 | 0.929 | 0.300 |

## e-Table 4: Adverse event prediction

**e-Table 4a**: Patient outcomes between 2015-2019 post-discharge, defining adverse events as death and readmission.

|  | 2015-2019 | | |
| --- | --- | --- | --- |
|  | **Death rate (%)** | **Readmission rate (%)** | **Adverse events rate (%)** |
| 24-hour | 1.22 | 2.24 | 3.45 |
| 48-hour | 1.86 | 3.40 | 5.19 |
| 72-hour | 2.34 | 4.30 | 6.55 |

**e-Table 4b**: Comparison of Adverse Events Post-Discharge from MICU in 2020 Based on Machine Learning Predictions of Patient Discharge Readiness (threshold 0.9) for Patients Discharged from the ICU in 2020 (Total N = 219)

|  | Models predict **discharge ready (n=89)** | | | Models predict **Not discharge ready (n=120)** | | |
| --- | --- | --- | --- | --- | --- | --- |
|  | Death | Readmission | Adverse events | Death | Readmission | Adverse events |
| 24-hour, n(%) | 0 | 3 (3.4%) | 3 (3.4%) | 1 (0.8%) | 9 (7.5%) | 10 (8.3%) |
| 48-hour, n(%) | 0 | 7 (3.4) | 7 (7.9%) | 3 (2.5%) | 16 (13.3%) | 19 (15.8%) |
| 72-hour, n(%) | 0 | 8 (9%) | 8 (9%) | 6 (5%) | 20 (16.7%) | 25 (20.8%) |

**e-Table 4c**. Relative Risk (RR) for Adverse Outcomes at 48- and 72-hours Post-ICU Discharge Based on Model Predictions for Patients Discharged from the ICU in 2020 (Total N = 219)

|  | **Model Prediction** | |  |
| --- | --- | --- | --- |
|  | Not discharge-ready (N =120) | Discharge ready (N = 89) | RR (95% CI) |
| **Adverse outcome at:** |  |  |  |
| **48hr, n(%)** | 19 (15.83%) | 7 (7.87%) | 2.01 (0.88-4.58) |
| **72hr, n(%)** | 25 (20.83%) | 8 (8.99%) | 2.32 (1.1 – 4.9) |

## Model Calibration

Brier score: 0.254 (95%CI: 0.253-0.255).

**e-Figure 3.** Calibration Curve of the LightGBM Predictive Model for Patient Discharge: The calibration curve illustrates the relationship between predicted discharge probabilities and observed discharge probabilities for the model. The blue squares represent the calibration points for different probability bins, and the shaded area indicates the 95% confidence interval (CI). The dashed black line represents a perfectly calibrated model where predicted probabilities match observed probabilities. The model's calibration slope is 0.90, and the calibration intercept is 0.005, indicating a slight underestimation of probabilities. This curve highlights the model's performance in predicting ICU discharge without adverse outcomes.


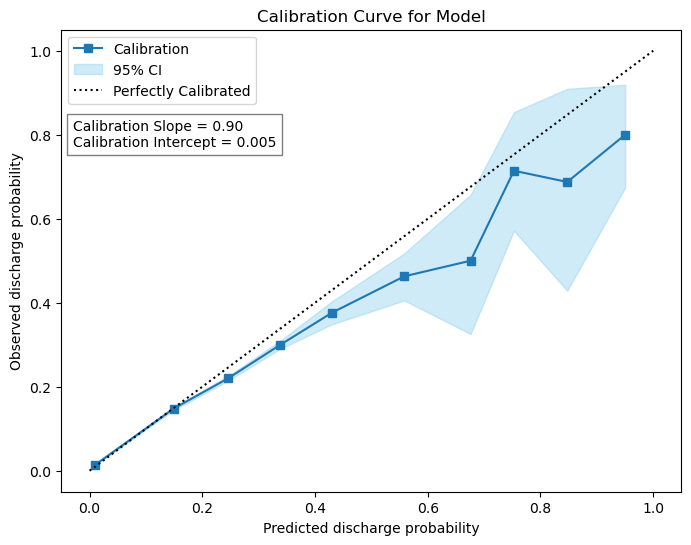


# Explainability analysis

e- **Figure 4.** The probability of readiness for discharge in different clinical features: (A) mean arterial pressures (MAP), (B) pulse, (C) respiratory rate, (D) hemoglobin, (E) Potassium, (F) Sodium


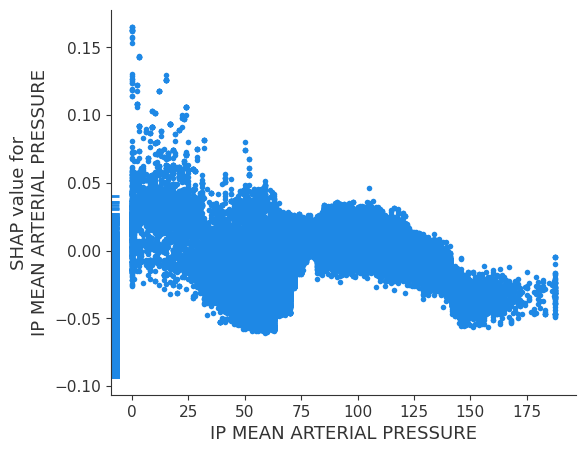

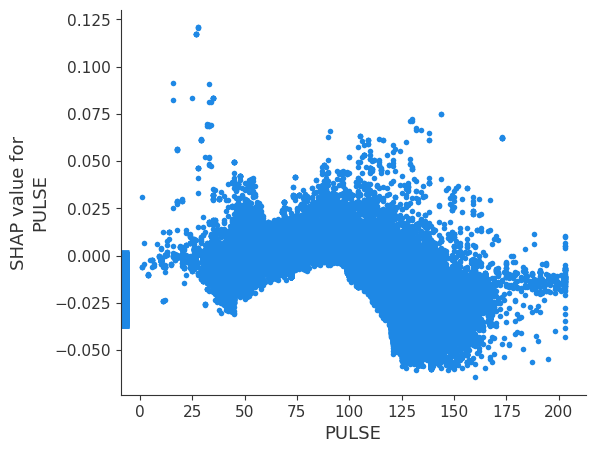

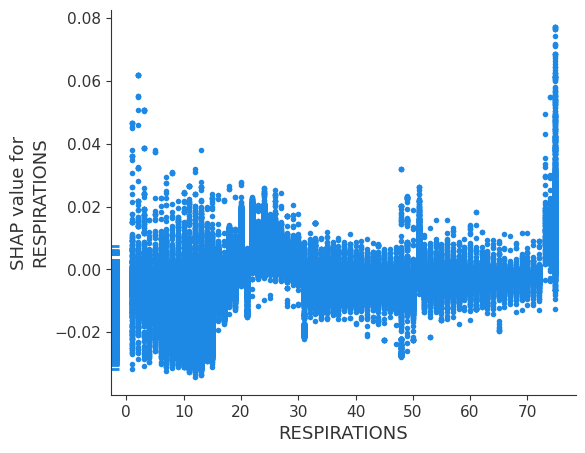

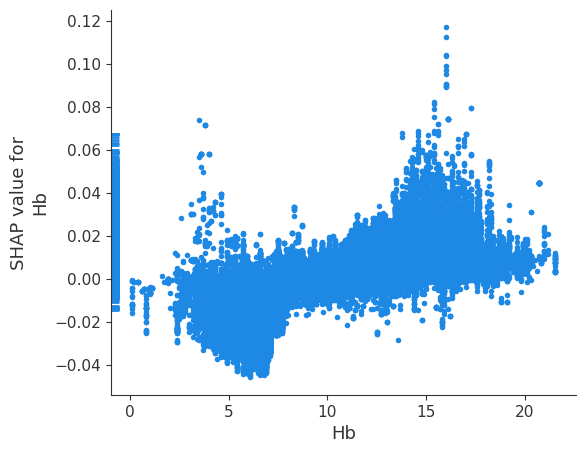

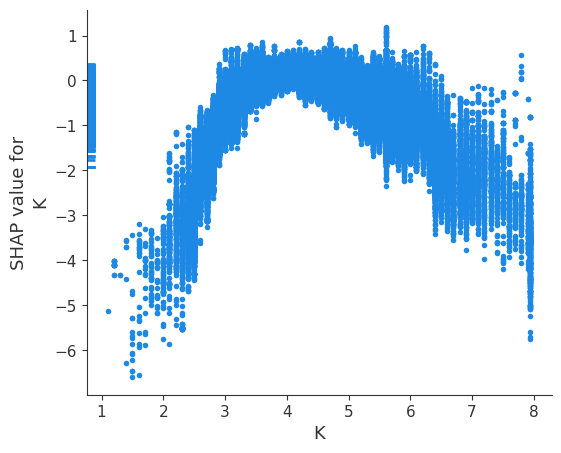

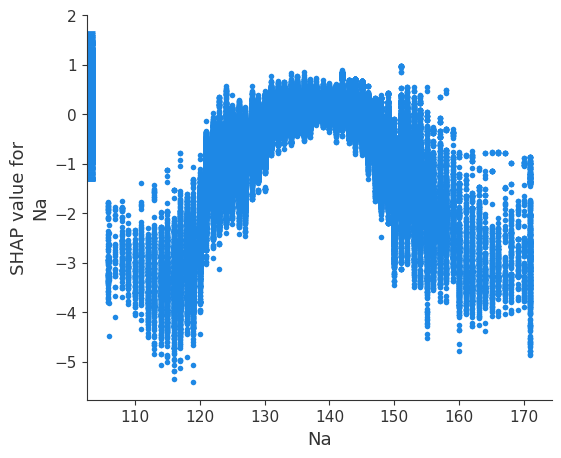


(F)

(E)

(D)

(C)

(B)

(A)

# User interface

**e-Figure 5**: At the time physician predict discharge at 8AM, model has predicted them ready for discharge for (a) **Threshold 0.9**: 5.0 (IQR: 2.0 – 13.5) consecutive hours, (b) **Threshold 0.85**: 9 (IQR: 3.0 – 17.0) consecutive hours.


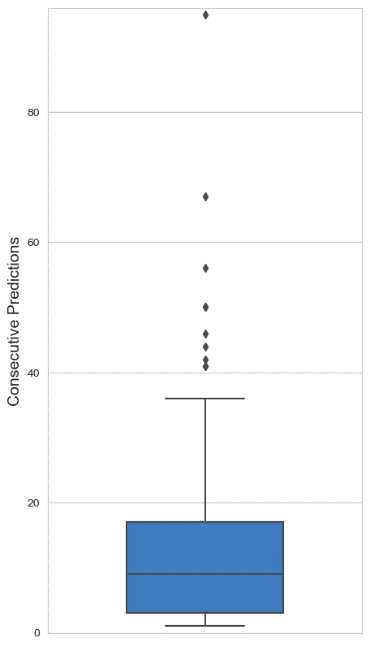

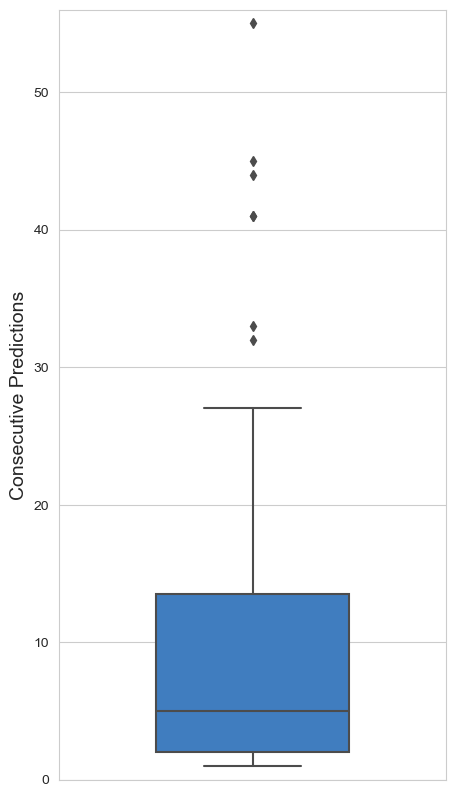


1. Threshold = 0.9
2. Threshold = 0.85

**e-Figure 6**. **Screenshot of ICU prediction model interface**. In the dashboard, provider can change the parameters and predict the patient's outcome. (A) Model prediction for patient #1 at admission, (B) Model prediction result after changing variables of O2 delivery method and length of stay.

**e-figure 6 (A)**


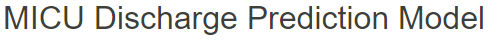


**
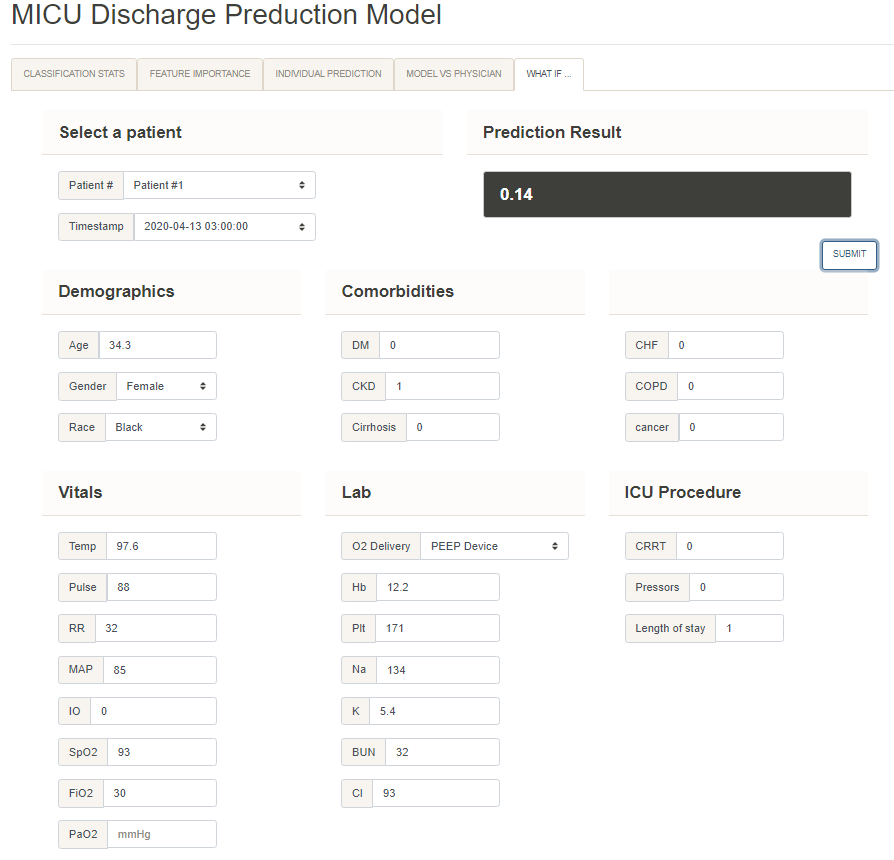
**

**e-figure 6 (B)**


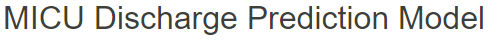


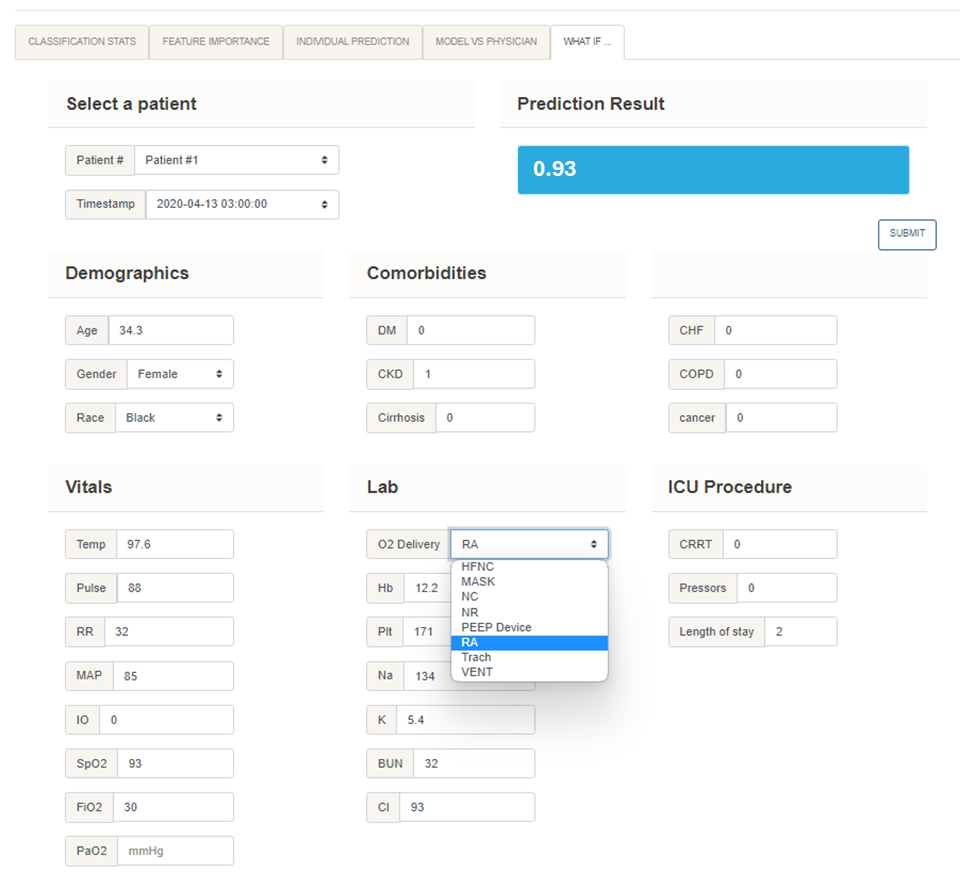


# Literature Search

We searched PubMed database for the following term: ((ICU) AND (discharge) AND (machine learning)) OR ((ICU) AND (discharge) AND (artificial intelligence)) to obtain a list of publications similar to ours. This list of articles was then screened manually by authors AB and CPW to see if the work published was original research reporting a prediction model for ICU discharge. Also, any article that was not published in English was not considered in our review.
